# Transactional leadership and intellectual capital, the mediating role of knowledge sharing: The study of customs employees in Sulaymaniyah governorate

**DOI:** 10.1016/j.heliyon.2024.e38747

**Published:** 2024-09-30

**Authors:** Doste Khoshnaw, Georgiana Karadaş

**Affiliations:** a Business Administration, Faculty of Economics and Administrative Sciences, Cyprus International University, Lefkosa via Mersin 10, Turkey; b Business Administration Department, Komar University of Science and Technology, Sulaimani, 46001, Kurdistan Region, Iraq

**Keywords:** Transactional leadership, Knowledge sharing, And intellectual capital

## Abstract

This study aims to examine the impact of transactional leadership style on intellectual capital, and knowledge sharing as a mediator between transactional leadership and each of the intellectual capital components (human capital, structural capital, and relational capital) in the public sector, in Sulaymaniyah governorate in north of Iraq. The research model was settled based on the previous investigations on transactional leadership, knowledge sharing, and intellectual capital. To collect the data, convenience sampling was utilized, questionnaires were sent to employees in five customs directorates, and 355 responses were received. The research model analytical estimation was conducted through the employment of (SEM) Structural Equation Modeling by using Partial Least Square (PLS). The findings of the research show that transactional leadership has a significant relationship with knowledge sharing as well as with all three components of intellectual capital. Knowledge sharing also has a significant correlation with the components of intellectual capital. Moreover, the study's results show that knowledge sharing mediates the relationship between transactional leadership with human capital, structural capital, and relational capital.

## 1 Introduction

Despite its significant role in all economies, the public sector has often been disregarded in intellectual capital studies. It's unexpected, especially given today's rapidly advancing, tech-centric, and knowledge-oriented landscape. Essentially, intellectual capital encompasses a company's innovative capabilities, accumulated experience, knowledge reservoir, client relationships, and expertise of its employees [1,2]. Public institutions have to understand that the only viable way to navigate this competitive environment is by building stronger, more effective organizations capable of developing firm-specific strategic assets [3]. Consequently, managing intellectual capital has become a paramount responsibility within an organization's administrative framework, especially in public sector [4].

Intellectual capital is a resource that facilitates long-term progress. It consists of human capital, structural capital, and relational capital [5]. Consistent with a firm's resource-based perspective [6], it emphasizes maintaining competitive strategies through using resources already in the organization. Resources must have certain qualities, such as being distinct, incomparable, and exceptional. These qualities can be seen in the experiences and skills that employees accumulate over time, as well as in the organizational process. Such internal resources can generate prosperity and are seen as incorporeal assets or intellectual capital, which means that a strategic resource is broadly acknowledged as a driving force behind business growth [7].

Adopting a knowledge management framework is one strategy for managing intellectual capital in the public sector. According to Ref. [8], an essential process in knowledge management is knowledge sharing, a strategic activity that adds value within organizational frameworks. This process necessitates comprehension, adaptation, and integration for effective implementation. So, public sector institutions can identify and utilize their intellectual capital to enhance performance and accomplish their goals by implementing a knowledge management framework. As stated by Ref. [9] intellectual capital is understood as an intangible, non-financial asset pivotal for organizational advancement, deriving from the institution's knowledge-based values. This requires public sector organizations to offer staff members the chance to experiment with new concepts, learn from their mistakes, and acquire new skills and knowledge. It is crucial to remember that managing intellectual capital is an ongoing process rather than a one-time event.

Additionally, the academic corpus extensively examines leadership, encompassing its varied styles and its association with a spectrum of organizational factors. These scholarly endeavors shed light on the intricacies of how distinct leadership styles influence individuals, collective teams, and the overarching performance of public sector [10]. According to Ref. [11] the position of leadership has a significant effect on the organization's success and failure. As a result, prosperous leaders can improve the various stakeholders' well-being of the organization, particularly its owners. As stated by Ref. [12] leadership is one of the primary management functions of any organization that considers it. Moreover, leadership aids in the coordination of personnel, resources, and timing to accomplish established organizational goals. The leader's relationship with his or her followers is referred to as leadership. Consistent with [13] effective leaders are leaders who recognize the importance of the development of the staff and how it is essential to facilitate change. To guarantee the sustained growth of intellectual capital, many companies invest in employee development. According to Ref. [14] leadership has a significant impact on intangible assets. Because of this, it is regarded as an essential component in any organization. Leadership holds the human capital enterprises in the first place, and it somehow becomes an intellectual capital component when seen as a process for leadership development. As a result, leadership focuses an individual's attention on interpersonal relationships in addition to their behavior and resources. Leadership also indicates the beneficial relationship between intellectual capital and leadership. Here, leadership strengthens an organization's intellectual capital, and this maintains the organization's competitive edge over its market competitors [15].

In the knowledge-based time, knowledge serves as an essential cornerstone for organizational competitiveness, growth, and long-term viability. Fostering a culture of knowledge sharing between employees and incentivizing them to integrate their specialized talents in day-to-day tasks can bestow a distinct competitive advantage [16]. It is believed that when employees exchange knowledge, it has a significant impact on the performance of both private and public sector organizations [17]. Consequently, organizations striving to achieve a competitive advantage have recognized the significance of knowledge sharing [18]. In addition, knowledge sharing is intrinsically linked to approaches that sustain an organization's competitive advantage and core competencies [19].

The relationship between leadership styles and intellectual capital components in private organizations has been studied extensively all over the world, for example, Poland, Pakistan, Kuwait, Malaysia, and other countries. Very few investigations have explored transactional leadership toward the components of intellectual capital in public sectors. Research by Ref. [20] explored the transformational leadership towards intellectual capital in construction companies in Poland [21], studied strategic leadership towards intellectual capital in Kuwait industrial companies [22], investigated the spiritual leadership towards intellectual capital in the automobile manufacturing industry in Pakistan, as well as [23] investigated transactional leadership towards intellectual capital but in the service industry in Malaysia [24]. Studied leadership towards employees and suggested other stakeholders to be considered in future research, as well as using moderators or mediators suggested to be investigated among leadership and other concepts by Ref. [25]. In the public sector institutions, especially in the Kurdistan region research on transactional leadership, intellectual capital, and knowledge sharing is limited and this study aims to fill in this gap in the literature. Moreover, extensive research on the influence of transactional leadership on organizational outcomes is lacking and the growing recognition of intellectual capital as a critical asset for organizational success is scarce, therefore deeper insight regarding the specific mechanisms through which these elements interact within public sector, particularly in non-western contexts is essential. While the mediating role of knowledge sharing has been explored in various private sector settings, its impact within the public sector, and specifically among customs employees in regions like the Sulaymaniyah Governorate, remains underexplored. This study seeks to fill this gap by examining how transactional leadership influences intellectual capital through the mediating role of knowledge sharing among customs employees in this unique geographical and organizational context.

## 2 Literature review

### 2.1 Transactional leadership

Transactional leadership is recognized as the interactive_relationship between leaders and their followers. This relationship directs followers concerning objectives by refining tasks and contingent rewards and punishments [26]. This leadership style motivates team members primarily through rewards (contingent rewards) and intervenes only when necessary (management by exception) [27]. According to Ref. [28], transactional leader applies rewards to ensure employees fulfill their job duties and implement punishments to adjust any abnormal behavior in the workplace. Transactional leadership can be described as an operational approach that emphasizes the use of rewards to motivate followers.

Contingent rewards can be seen as a positive transaction. Leaders delegate responsibilities to followers or mutually agree upon specific objectives with them. Subsequently, they ensure the provision of either psychological or material incentives for followers in exchange for the satisfactory completion of the designated task [29]. 'Management by exception' details how leaders either proactively address potential challenges (active management) or reactively manage them once they occur (passive management) [30].

### 2.2 Intellectual capital

The term "intellectual capital" has been defined in numerous ways [31]. Defined as the organization's intangible assets that contribute to its value, competitiveness, and sustainability. Intellectual capital is defined as the mixture of human, information, as well as organizational capital [32]. [9] Viewed intellectual capital as an intangible asset or organization's knowledge resource, facilitating the augmentation of revenue streams, enticing clientele, pioneering novel product developments, enhancing extant offerings, and furthering organizational advancement. At its core, intellectual capital is regarded as the collective body of knowledge or the intangible resource compilation that organizations know how to arrange to enhance their operations efficiently [33]. Intellectual capital is a complex concept combining experiences, knowledge, and skills essential to add value to an institution. It's an intangible asset crucial for organizational growth by deriving knowledge-based value [34]. [35] Notices that intellectual capital contains intangible assets and knowledge sourced that can be leveraged to forge new strategies, techniques, products, and services. Within any organization, this form of capital stands as a fundamental asset that stimulates innovation elevates operational prowess, and bolsters its position in the competitive landscape. According to Ref. [4], the three main intellectual capital components are human capital a set of skills, expertise, and knowledge of individuals. When the employee leaves the organization, human capital leaves. Structural capital refers to an organization's systems, structures, and procedures in an organization; and customer capital denotes the relationships and value derived from an organization's clientele. Human capital encompasses elements such as technical acumen, technological knowledge, technical expertise, educational background, professional qualifications, education-focused time, work of cherished values, and psychological assessment [36]. According to Ref. [37], structural capital relates to an organization's enduring elements that persist even after all personnel depart. The notion of structural capital parallels that of an organizational structure. It guarantees the company's continuity and advancement towards its objectives, even when interfacing with external factors. Essentially, structural capital significantly influences the organization's value. Relational capital pertains to the insights derived from the relation with any stakeholders internally or externally, that impacts the organization's trajectory and its capacity for value creation. This includes ties with customers, staff, both public and private collaborators, suppliers, and investors [38].

### 2.3 Knowledge sharing

Knowledge is a combination of contextual information, experiences, core values, expert insights, and intuitive understanding. Knowledge sharing involves exchanging information, offering feedback, analyzing mistakes, and determining the most effective methods for accomplishing a task [39]. This serves as a foundation to integrate new experiences with existing data. Knowledge sharing is a cultural practice rooted in social interaction, facilitating the dissemination of employees' knowledge, expertise, and skills across all organizational departments. It is not just a bidirectional exchange between those providing and receiving knowledge; the behavior of the knowledge provider predominantly influences it [19,40]. According to Ref. [41], the advantages of knowledge sharing include expanding networks, unlocking business opportunities, and refining processes for product and service development. Furthermore, knowledge sharing is described as the sharing information willingness of employees, which includes facts, processes, ideas, experiences, and formulas, with the other organization members [42].

### 2.4 Transactional leadership and intellectual capital

Effective leaders recognize the importance of employee growth as essential for facilitating and enabling change requirements. Many organizations commit to staff development to ensure sustainable growth of intellectual capital [13]. Leadership plays a pivotal role in influencing intangible resources. Consequently, it is seen as a vital component within any entity. At its core, leadership is integral to managing human capital. When viewed as a mechanism for nurturing leadership qualities, it becomes an integral part of intellectual capital. Leadership underscores the significance of interpersonal interactions, behavior, and capital. Furthermore, there exists a constructive relationship between leadership and intellectual capital. In this context, effective leadership augments an organization's intellectual capital, leading to ensuring a distinct advantage [15,43].

In many organizations, the transactional leadership style emerges as the most common leadership approach [44]. It has been highlighted that these leaders enhance the skills of their employees in problem analysis and resolution within the organization. By doing so, leaders foster employee professional development, which subsequently contributes to realizing the advantages of human capital [45]. It has been observed by Ref. [46] that these leaders often implement necessary cultural shifts to align organizational values. Crucially, leaders should provide insights into various performance facets of their team members, especially those influencing self-confidence, increasing job contentment, and pinpointing growth areas. Furthermore, such feedback can elevate job efficacy, attitudes within the organization, individual autonomy, self-recognition, dedication, self-worth, learning prospects, development, and the overall enhancement of human capital. According to Ref. [43], the correlation between transactional leadership and intellectual capital is both positive and significant. The impact of leadership on intangible assets has been widely acknowledged. The critical role of leadership as a component in organizational structure is widely acknowledged. The pivotal role of leadership in human capital enterprises cannot be overstated. When viewed as a process aimed at enhancing leadership skills, leadership assumes an integral component of an organization's intellectual capital framework. Consistent with [47], transactional leadership that employs a positive and active reward system, predicated on transactions between the leader and employees, effectively communicates expectations of attaining rewards. This practice leads to a constructive fostering of employees' creative behavior and overall satisfaction. As well [48], revealed that transactional leaders tend to foster particular human capital development, which is one of the intellectual capital components. So, based on what is argued above these hypotheses are stated.

H1a Transactional Leadership has a positive and significant impact on human capital.

H1b Transactional Leadership has a positive and significant impact on structural capital.

H1c Transactional Leadership has a positive and significant impact on relational capital.

### 2.5 Transactional leadership and knowledge sharing

The leaders perform within an organization [49]. The transactional leadership approach involves a thorough explanation of performance criteria for staff members, followed by the provision of motivation to encourage optimal outcomes that align with their interests in the attainment of overall objectives [50]. Transactional leadership involves explicitly articulating and reinforcing performance expectations for employees while simultaneously precluding any actions that fall outside of the established agreement. The transactional leader is known to facilitate employees’ compliance by offering significant rewards and punishments [44]. According to Ref. [51], the incentivization of monetary rewards and recognition, as employed by transactional leadership within an organization, appears to have a positive impact on the dissemination of knowledge within said organization. In conjunction with the study by Ref. [52], it should be noted that the provision of contingent rewards by transactional leaders has a significant impact on the promotion and sustenance of exploitative innovation within an organizational setting. Moreover, the fiscal incentives and acknowledgments emanating from a transactional leadership style serve to incentivize and drive performance. The act of sharing knowledge within an organizational context is a crucial aspect of its overall functioning and success [49]. Transactional leaders undertake a critical part in knowledge-sharing management effectively within an organizational context. Studies by Refs. [49,53], and [54] have demonstrated that transactional leadership exerts a significant effect on the knowledge-sharing process. Based on what was discussed and mentioned above the hypothesis below is stated.

H2 Transactional Leadership has a significant and positive impact on knowledge sharing.

### 2.6 Knowledge sharing and intellectual capital

The extension of the firm's resource-based theory is the firm's knowledge-based theory. The knowledge-based theory concentrates on the embedded individual employee's knowledge and the overall knowledge of the firm as a competitive advantage source [55,56]. This perspective posits that a firm's primary competitive edge originates from its internal resources and capabilities. According to this theory, a company's assets, encompassing both tangible and intangible elements, play a pivotal role in driving its performance and competitive stance. For optimal outcomes, these assets should be leveraged both effectively and efficiently in executing the firm's unique and lucrative strategies, moreover, knowledge possesses the most strategic meaning in an organization [16,19]. According to Refs. [57,58], The Knowledge-Based View proposes that knowledge possession serves as the foremost foundation of value, whilst the creation of value for an organization is fundamentally contingent on its capability to acquire, utilize, and share knowledge. The firm's competitive advantage source is not knowledge, it is integrated knowledge. The knowledge heterogeneity problem-solving inside the firm is creating collective knowledge from individual knowledge [59]. Stated differently, it is hard for unintegrated knowledge to generate genuine intellectual capital. Furthermore, the knowledge sharing extent would influence the knowledge integration effectiveness [60].

[31] defines intellectual capital as the “Stock of Knowledge” owned by an organization and encompasses human, structural, and relational capital. Intellectual capital is typically understood as assets that organizations utilize to achieve and continue the competitive edge. These assets are typically based on knowledge acquisition and utilization [61]. Knowledge sharing affects human, structural, and relational capital significantly. It increases the depth of intellectual capital, expands one's individual skills and knowledge awareness, as well as increases the other's application capability. As a consequence of frequent sharing and practice, optimized knowledge is enhanced in the organization, and trust is strengthened [62].

Knowledge sharing is vital for an organization's long-term success and competitive advantage. Intellectual capital, which encompasses expertise, innovation, systems, and relationships, defines a company's competitive position. These assets are categorized into human, structural, and relational components. The organization's strength lies in its skilled personnel capable of problem-solving and decision-making, with knowledge sharing bolstering this human resource potential. As well as by fostering knowledge transfer across all employee levels, businesses can consistently uphold product and service quality. Such knowledge exchange fortifies lasting relationships with customers [19]. In an organization, team members often possess unique skills that, when combined, enhance overall performance and interpersonal bonds. Collaborative efforts and knowledge exchange foster a stronger organizational structure, marked by frequent interactions and mutual trust [63]. Knowledge sharing accommodates humans, procedures, and technology to attain continuous competitive advantage [64]. Since intellectual capital focuses on knowledge and because knowledge sharing is the transferring of knowledge, as well as knowledge sharing raises intellectual capital [65], knowledge sharing should be used to grow intellectual capital.

Along with the reviewed literature hypothesis 3a,3b,3c are drafted as.

H3a Knowledge sharing has a positive and significant impact on human capital.

H3b Knowledge sharing has a positive and significant impact on structural capital.

H3c Knowledge sharing has a positive and significant impact on relational capital.

### 2.7 Knowledge sharing the mediating role

As [66] stated intellectual capital is a “packaged useful knowledge”. As well as, because the evolution of intellectual capital within an organizational setting was shaped by the outcomes arising from knowledge management processes [67]. Along with the above-mentioned research, extensive scholarly investigations examining the drivers of knowledge-sharing have consistently emphasized the essential role of leadership in enabling and fostering knowledge-sharing endeavors [68]. It can be hypothesized that knowledge sharing has a mediating role between transactional leadership style and intellectual capital since the empirical findings by Ref. [69] have yielded compelling and substantial evidence underscoring knowledge sharing's pivotal role as a mediating factor within the examined context. These empirical results contribute valuable comprehension of the intricate dynamics of knowledge sharing and its influence on various aspects of organizational processes, performance, and outcomes. By serving as a mediator, knowledge sharing enables the transfer, integration, and dissemination of valuable knowledge and information, thereby enhancing communication, collaboration, and decision-making processes. This empirical evidence further highlights the significance of knowledge sharing as a mechanism that mediates the relationship among relevant variables, offering a comprehensive understanding of its impact on organizational performance and effectiveness. A study conducted by Ref. [70] examined the adjusted function of knowledge sharing in the relationship concerning human capital and job empowerment at Jadara University. The study found a positive and statistically significant influence of knowledge sharing on the association between human capital and empowerment at Jadara University. The study by Ref. [21] revealed a strong and statistically significant correlation between different leadership styles concerning intellectual capital and the mediating role of knowledge sharing among industrial companies operating in the State of Kuwait. These findings underscore the significance of strategic leadership practices in effectively managing intellectual capital and provide insights into the crucial role played by knowledge sharing as a mechanism through which the benefits of strategic leadership are realized within the specific context of industrial organizations in Kuwait.

Based on the above-mentioned arguments and findings regarding transactional leadership and knowledge sharing, it is hypothesized that both transactional leadership and knowledge sharing have a positive and significant relation with all three components of intellectual capital. Furthermore, transactional leadership is hypothesized to also have a positive and significant relation with knowledge sharing. Therefore, the following hypotheses are put forward.

H4a Knowledge sharing has a mediating role between transactional leadership and human capital.

H4b Knowledge sharing has a mediating role between transactional leadership and structural capital.

H4c Knowledge sharing has a mediating role between transactional leadership and relational capital.

**Fig. 1:**
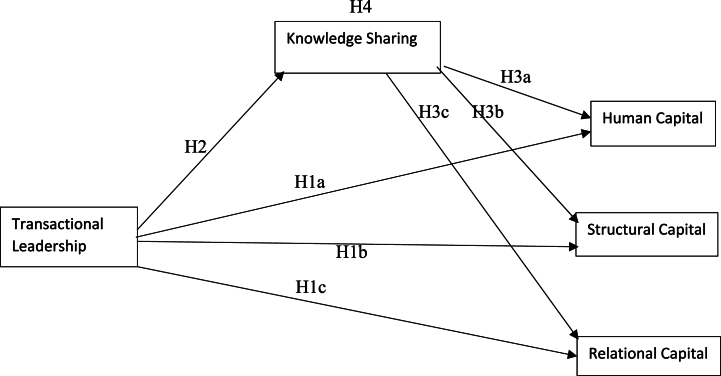
The model.

Based on the discusion and crafting hypothesis above the research model is created as shown in (Fig. 1).

### 2.8 Methodology

This investigation followed the quantitative method and the population consists of all employees working in directorates of customs in Sulaymaniyah Governorate in the Kurdistan Region of Iraq, There are 1076 employees distributed to five directorates as shown in Table 1.

**Table 1:** The number of employees in each directorate.

Directorate name	Number of employees
Sulaymaniyah customs directorate	333
Customs directorate of Bashmax International Port	207
Customs directorate of Parwesxan international port	222
Pshdar customs directorate	184
Customs directorate of Sulaymaniyah international airport (specialized in Air cargo)	130
Total	1076

Convenience sampling was implemented to collect data via an online questionnaire. The questionnaire was translated from English into the Kurdish Language to be more clear for respondents. Convenience sampling entails choosing participants based on their immediate accessibility to the researcher. Using this approach, the researcher picks individuals due to their proximity, without assessing whether they accurately represent the broader population [71]. The questionnaire was filled up by 355 employees in five different directorates. A total of 107 employees completed the survey from the Sulaymaniyah customs directorate, 67 employees from the Customs directorate of Bashmax International Port, 72 responded from the Customs directorate of Parwesxan International Port, 68 employees from the Pshdar customs directorate and 41 employees from the Customs directorate of Sulaymaniyah international airport, respectively.

All measures in the investigation were based on a 5-Likert scale except the measurement of transactional leadership which was measured by a 7-Likert scale. Five items to measure transactional leadership were adopted, that is multi-factor leadership measure was used [72]. Employees were asked about their leader's behavior for instance, “My leader monitors my performance and keeps track of mistakes”. Six items to measure knowledge sharing were adopted [73]. Employees were asked about knowledge sharing among each other. Such as, “I try to share my expertise from education or training with other group members more effectively”. Intellectual capital consisting of fifteen items was adopted [63], each of the three dimensions of intellectual capital has its questions. Five items for each dimension of intellectual capital including human, structural, and relational capital were implemented [63].

The demographic questions were related to gender, age, years of experience, and employee grades are categorized into five categories, each one of which has different ranges based on the nature of the question. The questionnaire was approved by the ethical committee in their meeting EKK23-24/004/002 on December 27th, 2023, with a formal letter numbered 020–382 on January 15th, 2024.

### 2.9 Data analysis and findings

Table 2 provides an overview of the population's demographics, with 72.1 % being male, highlighting a notable gender imbalance as females constitute less than one-third. According to the age of participants, 59.4 % of participants are aged 28–37, with fewer in older groups and only 1.1 % in the 18–27 years old. Furthermore, 52.1 % have a bachelor's degree, while just 2.5 % have a higher qualification. Moreover, 62.5 % of participants have 8–14 years of work experience, with only 7.3 % having 29 years or more. 70 % of employees fall into two Grade categories 8-7 and 6-5, with the fewest in Grade 10-9 at 5.6 %. The dataset mainly consists of mid-career, predominantly holding bachelor's degrees and higher than bachelor has the fewest share.

**Table 2:** Demographic analysis.

Category	Category	Frequency	Percentage
Gender	male	256	72.10
	female	99	27.90
	total	355	100
Age	18–27	4	1.1
	28–37	211	59.4
	38–47	85	23.9
	48–57	38	10.7
	58 and older	17	4.8
	total	355	100
Level of education	elementary school	28	7.9
	high school	31	8.7
	associate degree	102	28.7
	bachelor degree	185	52.1
	above bachelor	9	2.5
	total	355	100
Experience	1–7 years	29	8.2
	8–14 years	222	62.5
	15–21 years	43	12.1
	22–28 years	35	9.9
	29 years and more	26	7.3
	total	355	100
Employee grade	Grades 10 & 9	20	5.6
	Grades 8 & 7	128	36.1
	Grades 6 & 5	121	34.1
	Grades 4 & 3	48	13.5
	Grades 2 &1	38	10.7
	total	355	100

The data was analyzed using second-generation multivariate PLS-SEM, as outlined by Ref. [74]. PLS-SME is well-suited to forecast and adapt handling complicated models with multiple structural relations. It provides precise estimations when dealing with large sample sizes, accommodates both multi-item and single measures as well, along competently works with reflexive as well as formative measurement models [75]. There are no missing values reported in the study. The complete 355 responses are used for analysis.

**Fig. 2:**
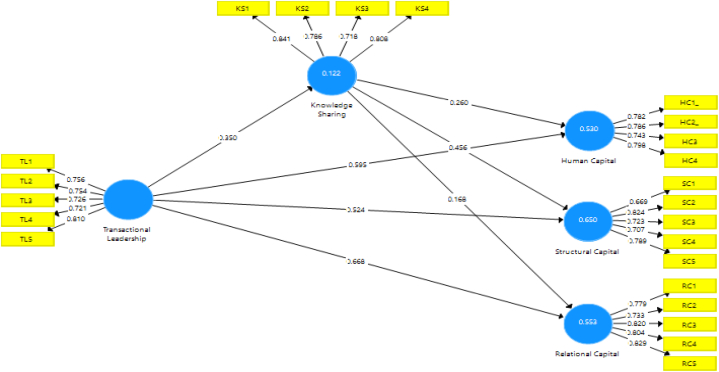
Measurement model.

In this study, all latent variables were treated as reflective. The measurement model was employed to estimate the reliability and validity of the measures as shown in (Fig. 2). Specifically, the outer loading of all study composes were examined to assess their reliability indicator. It was found that each item's standardized outer loading value exceeded 0.50, indicating satisfactory reliability according to Ref. [75]. The high outer loading values observed in this study surpassed the established threshold value, ensuring the reliability of the measures used but if 2 dimensions of the knowledge-sharing leading are less than the standard then it is excluded and only four dimensions are fulfilling the PLS criteria for estimation.

To measure the internal consistency of the measures the value of Cronbach's alpha was assessed. To establish robust internal consistency, the alpha value should exceed the threshold of 0.70. The results, displayed in Table 3, revealed that the values of Cronbach alpha fell within the range of 0.764–0.920, demonstrating a strong level of internal consistency in the current study.

**Table 3:** Assessment of reflective model.

First Order Construct	Items	Loading	α	Composite Reliability	AVE
**Human Capital**	**HC1_**	0.782	0.782	0.859	0.605
	**HC2_**	0.786			
	**HC3**	0.743			
	**HC4**	0.798			
**Knowledge Sharing**	**KS1**	0.841	0.798	0.868	0.623
	**KS2**	0.786			
	**KS3**	0.718			
	**KS4**	0.808			
**Relational Capital**	**RC1**	0.779	0.853	0.895	0.630
	**RC2**	0.733			
	**RC3**	0.820			
	**RC4**	0.804			
	**RC5**	0.829			
**Structural Capital**	**SC1**	0.669	0.798	0.861	0.554
	**SC2**	0.824			
	**SC3**	0.723			
	**SC4**	0.707			
	**SC5**	0.789			
**Transactional Leadership**	**TL1**	0.517	0.810	0.868	0.568
	**TL2**	0.734			
	**TL3**	0.784			
	**TL4**	0.758			
	**TL5**	0.786			

Additionally, composite reliability was employed as an additional standard for assessing the internal consistency reliability. For composite reliability, a coefficient higher than 0.70 is considered acceptable. The composite reliability values exceeded 0.70 as shown in Table 3, demonstrating a decent and reliable internal consistency level across the measures.

To assess convergent validity, the recommended approach was to utilize the average variance extracted (AVE), as proposed by Ref. [76]. Convergent validity pertains to what way a measure connects to other measurements of the variables. The AVE and outer loading values of all studied constructs were assessed to estimate convergent validity. AVE values should exceed 0.50 [75]. The findings of this study illustrate that values of AVE surpassed the threshold of 0.50, thus confirming the establishment of convergent validity.

Furthermore, to ensure that each studied construct is distinct from others, the evaluation of construct validity was done [77]. To accomplish this, recommended methods included the Fornell-Larcker test [76], heterotrait–monotrait (HTMT) ratio [78], as well as cross-loadings analysis. These techniques were employed to validate and distinguish the studied constructs from one another.

The Fornell-Larcker test was conducted to evaluate discriminant validity. Following this test, the average variance extracted (AVE) square root should exceed all other constructs’ correlations. The results, as shown in Table 4, clearly indicate that discriminant validity was successfully established.

**Table 4:** Fornell-Larcker criterion.

SR	Variables	Human Capital_	Knowledge Sharing	Relational Capital_	Structural Capital	Transactional Leadership
**1**	**Human Capital_**	0.778				
**2**	**Knowledge Sharing**	0.468	0.790			
**3**	**Relational Capital_**	0.771	0.402	0.794		
**4**	**Structural Capital**	0.819	0.640	0.769	0.744	
**5**	**Transactional Leadership**	0.686	0.350	0.726	0.684	0.754

The second point of comparison for correlation assessment between endogenous variables is the HTMT (heterotrait-monotrait) ratio, which is recommended for determining if a variable is distinct from others [79]. When the HTMT value is below 1, it indicates that the variable exhibits discriminant validity. Upon examining all the values presented in Table 5, it is evident that they were below 0.90. As a result, it can be decided that discriminant validity has been effectively proven.

**Table 5:** HTMT criterion.

		Human Capital_	Knowledge Sharing	Relational Capital_	Structural Capital	Transactional Leadership
1	**Human Capital_**					
2	**Knowledge Sharing**	0.593				
3	**Relational Capital_**	0.943	0.483			
4	**Structural Capital**	1.038	0.787	0.932		
5	**Transactional Leadership**	0.860	0.431	0.873	0.851	

Another criterion utilized for evaluating discriminant validity involves comparing item loadings to item cross-loadings [80]. In the present study, the findings show that the items’ loading values were greater than their respective cross-loading values, as demonstrated in Table 6. As a result, it can be confidently stated that discriminant validity has been successfully established.

**Table 6:** Cross loading.

	Human Capital_	Knowledge Sharing	Relational Capital_	Structural Capital	Transactional Leadership
**HC1_**	0.782	0.450	0.600	0.674	0.475
**HC2_**	0.786	0.354	0.632	0.652	0.596
**HC3**	0.743	0.339	0.571	0.602	0.515
**HC4**	0.798	0.316	0.592	0.617	0.541
**KS1**	0.380	0.841	0.328	0.544	0.273
**KS2**	0.402	0.786	0.357	0.523	0.335
**KS3**	0.351	0.718	0.243	0.449	0.217
**KS4**	0.343	0.808	0.330	0.496	0.270
**RC1**	0.577	0.232	0.779	0.551	0.571
**RC2**	0.595	0.348	0.733	0.610	0.512
**RC3**	0.621	0.325	0.820	0.631	0.614
**RC4**	0.622	0.347	0.804	0.628	0.592
**RC5**	0.644	0.341	0.829	0.630	0.589
**SC1**	0.574	0.325	0.503	0.669	0.454
**SC2**	0.664	0.604	0.605	0.824	0.540
**SC3**	0.574	0.475	0.562	0.723	0.502
**SC4**	0.644	0.439	0.556	0.707	0.524
**SC5**	0.593	0.498	0.628	0.789	0.523
**TL1**	0.548	0.247	0.520	0.515	0.756
**TL2**	0.508	0.269	0.514	0.494	0.754
**TL3**	0.495	0.203	0.551	0.527	0.726
**TL4**	0.491	0.290	0.548	0.478	0.721
**TL5**	0.544	0.307	0.602	0.561	0.810

In the subsequent step, the structural model is evaluated using a bootstrapping method. Initially, the presence of collinearity was checked when estimating the structural model. Collinearity deals with a high correlation among the variables under study [81]. The standard measure for collinearity detection is the variance inflation factor. To avoid collinearity issues, the VIF values were expected to not reach 5. Upon analysis, the findings indicated that the VIF value ranges were between 1.72 and 2.31, indicating the absence of collinearity in our data.

Moving forward, the path coefficient representing the hypothesized linkages was assessed using the PLS algorithm. The determination of the significance of these coefficients was made using bootstrap tests, where t-statistics exceeding 1.96 (p < 0.05) indicating the significance of the relationship.

Subsequently, to assess the extent of variance explained by exogenous variables the determination coefficient (R2) was calculated. The values of (R2) are represented in Table 7, indicating the extent of variance explained. Following the recommendations by Ref. [81], standard (R2) values of 0.75 (substantial), 0.50 (moderate), and 0.25 (weak) were considered to interpret the extent of explained variance.

**Table 7:** Structural model Fitness summary.

	R_2_	Sample Mean (M)	Standard Deviation (STDEV)	T Statistics	P Values
Human Capital	0.530	0.535	0.047	11.341	0.000
Knowledge Sharing	0.122	0.127	0.040	3.063	0.002
Relational Capital	0.553	0.555	0.048	11.549	0.000
Structural Capital	0.650	0.654	0.037	17.505	0.000

**Fig. 3:**
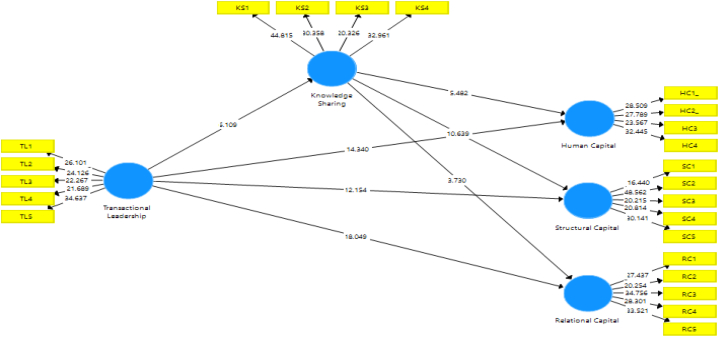
Structural Equation model.

The results in Table 8 demonstrate the significant relation between transactional leadership and knowledge sharing at a 1 % level of significance. The findings show that every hypothesis is strongly supported, with p-values of 0.000 for all the proposed relationships, indicating that they are all statistically significant. Human capital (confidence interval: 0.170–0.351), relational capital (0.078–0.258), and structural capital (0.372–0.532) are all significantly increased by knowledge exchange with the effect of 0.47,0.45 and 0.43, respectively. Relational capital (0.587–0.732), knowledge sharing (0.235–0.459), human capital (0.513–0.673), and structural capital (0.440–0.608) are all positively impacted by transactional leadership with the effect of 0.57, 0.37 and 0.43. Furthermore, through knowledge sharing, transactional leadership affects relational capital (0.652–0.871), structural capital (0.243–0.652), and human capital (0.723–0.651). These findings highlight the value of transactional leadership and knowledge sharing in building intellectual capital. Moreover, based on the study findings it becomes apparent in the structural equation model (Fig. 3) that knowledge sharing has a mediating role between transactional leadership and relational, structural, and human capital.

**Table 8:** Results findings and hypothesis testing.

	Effect (f^2^)	T Statistics	P Values	Decision	Confidence interval	
					2.50 %	97.50 %
Knowledge Sharing - > Human Capital_	0.047	5.482	0.000	Supported	0.170	0.351
Knowledge Sharing - > Relational Capital_	0.045	3.730	0.000	Supported	0.078	0.258
Knowledge Sharing - > Structural Capital	0.043	10.639	0.000	Supported	0.372	0.532
Transactional Leadership - > Human Capital_	0.041	14.340	0.000	Supported	0.513	0.673
Transactional Leadership - > Knowledge Sharing	0.057	6.109	0.000	Supported	0.235	0.459
Transactional Leadership - > Relational Capital_	0.037	18.049	0.000	Supported	0.587	0.732
Transactional Leadership - > Structural Capital	0.043	12.154	0.000	Supported	0.440	0.608
Transactional Leadership - > Knowledge Sharing- > Human Capital	0.067	20.365	0.000	Supported	0.723	0.651
Transactional Leadership - > Knowledge Sharing- > Structural Capital	0.176	9.309	0.000	Supported	0.243	0.652
Transactional Leadership - > Knowledge Sharing- > Relational Capital	0.512	34.001	0.000	Supported	0.652	0.871

## 3 Discussion on findings

This investigation examines the relationship between transactional leadership and intellectual capital within public sector, with attention to the mediating role of knowledge sharing. It gives a further understanding of how transactional leadership influences intellectual capital and the role of knowledge sharing as an intermediary variable. The findings of this investigation can be summarized from three primary points of view.

First of all, transactional leadership, commonly observed among public sector leaders, significantly influences the dimensions of intellectual capital, as well as knowledge sharing. Our findings, supporting Hypotheses H1a, H1b, and H1c, suggest a significant positive impact of transactional leadership on the components of intellectual capital. In this way, our findings are consistent with studies by Refs. [82,83], and [43]. Leadership can be the main factor in creating and growing intellectual capital in organizations to achieve long-term success. The skills and knowledge that an organization's employees possess is the intellectual capital. To gain a competitive advantage through human capital, organizations must invest because this will contribute to developing the intellectual capital of the organization. As well as leaders are key characters in promoting new talents among employees on a single platform, motivating followers to accomplish common objectives, strengthening the individual's internal interest in their jobs, and helping retain them in the organization.

Further, the present results, in line with [16,54,84], and [85] reinforce Hypothesis H2 by illustrating transactional leadership's positive relation with knowledge sharing. As well as the leader's active and appropriate involvement can enforce good knowledge sharing because the leader is the most important individual who has an essential role in communicating with others and forming consciousness of the necessary knowledge to achieve the goal [86]. This means that transactional leaders may use contingent rewards to persuade employees toward knowledge sharing. Additionally, knowing that there is a reward for someone to share what he knows, increases the employee's willingness to share knowledge and reduces the fear that makes someone disinclined to knowledge sharing.

Secondly, the research findings support Hypotheses H3a, H3b, and H3c, highlighting the knowledge sharing's significant and positive relation with intellectual capital. This is supported by Ref. [63] [87], as well as [22]. When sharing expertise and knowledge has been implemented among employees, it will help them to develop their skills and learn more. New techniques will be gained as a consequence of knowledge sharing. Not only employees will benefit from knowledge sharing, but other stakeholders will also be served as well to develop their skills that is because it sets up an effective social bond between them.

Moreover, the current study's results indicate that knowledge sharing is a mediator between transactional leadership and intellectual capital components which means H4a, H4b, and H4c are supported, which is in line with [21,22,88]. A responsive environment enables employees to have free interaction, as well as sharing their views and helping others. Consequently, knowledge sharing will occur to help each other. Subsequently, knowledge sharing between employees will increase and a strong social connection will be enforced [89]. The knowledge will not be received just from inside the organization, suppliers and customers may be the source of learning for employees through exchanging views. Contingent reward from organizations drives employees through knowledge development by exchanging views with stakeholders and customers [90]. This will assist organizations to function better and establish a new system.

This research adds significantly to the body of knowledge on leadership and organizational behavior in the public sector. Initially, it broadens the comprehension of transactional leadership by showcasing its noteworthy enhancement of intellectual capital, thus emphasizing the part leadership plays in augmenting organizational knowledge [70]. Furthermore, the study highlights the significance of information sharing as a critical mediating factor, providing empirical proof that transactional leadership cultivates a situation that is favorable to knowledge sharing and cooperation [16]. This emphasizes how important it is for public sector organizations to fund leadership development initiatives that promote knowledge-sharing practices [84].

Finally, by concentrating on Sulaymaniyah Governorate customs workers, the study fills a gap in the literature by offering insightful information about the dynamics of knowledge management and leadership in a non-Western setting. This advances a more comprehensive understanding of these phenomena globally. These findings have implications for leaders in the public sector and policymakers who want to use intellectual capital to boost long-term success and organizational performance [22].

## 4 Conclusion

The study concludes that transactional leadership has significant impacts on intellectual capital in public sector organizations, especially when it comes to the mediating role that information exchange among customs officers in Sulaymaniyah Governorate plays. The results demonstrate that transactional leadership increases intellectual capital by creating a setting that encourages information exchange. Effective transactional leadership techniques, including contingent compensation, can help leaders foster collaboration and knowledge sharing, which will increase the intellectual capital of the company. This study emphasizes how important it is for leaders to foster a culture of information sharing in order to foster the growth and management of intellectual assets, which in turn improves organizational performance and long-term success.

This study was conducted in the entirely different context, and it is made by the call of [2] in order to expand the public sector intellectual capital research, and [43] in order to find out the effect of leadership style on intellectual capital within different context. This paper answers the call of [91] but to find out the transactional leadership's impact on structural capital, relational capital in addition to human capital, and [20] but by examining the impact of transactional leadership not transformational leadership on intellectual capital, but within different organizations, and even different context. This study investigated that transactional leadership effects all the three components of intellectual capital regarding the public sector organizations in the research context. Regarding the knowledge sharing the study reveals that knowledge sharing mediates the relation between transactional leadership and all three components of intellectual capital as it is clear in knowledge-based theory.

Leaders must be aware of the importance of staff development for successful intellectual capital growth [13]. So, this investigation can be regarded one of the first attempts within the context of public sector in Kurdistan Region of Iraq as a “developing country”. The implications of researching the relationship between transactional leadership and knowledge sharing, relational capital, and structural capital are varied and give organizations and leaders useful insights. Organizations develop more effective knowledge-sharing efforts if they understand the impact of transactional leadership on information sharing. Leaders may encourage people to contribute their knowledge and expertise more voluntarily by carefully using rewards and incentives, resulting in a more collaborative and learning-oriented culture. Ultimately, the implications of the study extend to the long-term success and competitiveness of the organization. A well-balanced approach to leadership and knowledge sharing can lead to the accumulation of intellectual capital, fostering innovation, and creating a resilient and agile organization capable of adapting to changing environments. Overall, the findings highlight the significant implications of leadership practices in developing an organization's knowledge-sharing and intellectual capital. Leaders who recognize and embrace their role in supporting information sharing and intellectual capital development can significantly contribute to the growth and success of their organization.

In a similar way, the practical implications of this study highlight the critical role of knowledge sharing and transactional leadership in organizational development. Organizations should foster a culture of knowledge sharing to enhance human, relational, and structural capital, which are vital for sustainable competitive advantage. Leaders who adopt transactional leadership practices can significantly boost these forms of capital both directly and indirectly through encouraging knowledge sharing. Implementing structured leadership training and knowledge management systems can thus lead to improved employee skills, stronger internal relationships, and more robust organizational structures, ultimately driving overall organizational performance and innovation. Moreovers, practical implications for public sector organizations include the need to implement structured transactional leadership frameworks that emphasize clear objectives, rewards, and feedback mechanisms to enhance employee motivation and performance. This leadership approach should be complemented by initiatives that encourage and facilitate knowledge sharing, such as creating collaborative platforms, offering training programs, and recognizing knowledge-sharing behaviors. By doing so, public sector organizations can harness the collective expertise of their workforce, leading to improved efficiency, innovation, and service delivery.

In every research endeavor, certain limitations exist, and this investigation is no exception. First and foremost, the findings are derived from cross-sectional data, constraining the depth of causality examinations. As such, our proposed framework aligns with the inherent directionality of previous studies. Secondly, this research chiefly examines the transactional leadership's influence on intellectual capital and knowledge sharing as well, and the role of knowledge sharing as a mediator. Although such a specific concentration facilitates a thorough analysis, it might inadvertently overlock other pivotal leadership styles especially bureaucratic leadership, which could present varied interrelationships with the observed variables. Diverse organizational and cultural contexts might modify the interplay among leadership styles, knowledge sharing, and intellectual capital. Future studies may investigate the wider sample size, other cities or other public sector organizations such as tax directorates, public sector banks. Future research may investigate bureaucratic leadership, authoritarian leadership instead of transactional leadership. Finally, the most potential sector to be investigated would be security sector.

## Data availability statement

The data are available upon the request from the corresponding author.

## CRediT authorship contribution statement

**Doste Khoshnaw:** Writing – review & editing, Writing – original draft. **Georgiana Karadaş:** Supervision.

## Declaration of competing interest

The authors declare that they have no known competing financial interests or personal relationships that could have appeared to influence the work reported in this paper.
