# Supplementary material for: Transactional leadership and intellectual capital, the mediating role of knowledge sharing: The study of customs employees in Sulaymaniyah governorate
Source: Heliyon. 2024 Sep 30;10(19):e38747. doi: 10.1016/j.heliyon.2024.e38747 (PMC11483484; doi:10.1016/j.heliyon.2024.e38747)
Supplement: Multimedia component 1 [file mmc1.docx]

Demographic Questions

Gender

1. Male
2. Female

Age

1. 18-27 years’ old
2. 28-37 years’ old
3. 38-47 years’ old
4. 48-57 years’ old
5. 58 years old or older

education level

1. Elementary school
2. High school
3. 2 years’ institute after high school
4. Bachelor’s degree
5. Higher than bachelors

work experience

1. 1-7 years
2. 8-14 years
3. 15-21 years
4. 22-28 years
5. 29 years or more

employee grade

1. Grade 10-9
2. Grade 8-7
3. Grade 6-5
4. Grade 4-3
5. Grade 2-1

For Transactional leadership

Please chose

1. If you are strongly disagree
2. If you are disagree
3. If you are Somewhat disagree
4. If you are Neither agree nor disagree
5. If you are somewhat agree
6. If you are agree
7. If you are strongly agree

**Transactional leadership**

TL1: my leader makes clear expectations

TL2: my leader will take action before problems are chronic

TL3: my leader tells us standards to carry out work

TL4: my leader works out agreement with me

TL5: my leader Monitors my performance and keeps track of mistake

For knowledge sharing and Intellectual capital

Please chose

1. If you are Strongly disagree
2. If you are Disagree
3. If you are Neither disagree nor agree
4. If you are Agree
5. If you are Strongly agree

**Knowledge Sharing**

KS1: I actively share my professional knowledge with my colleagues

KS2: I voluntarily share my skills with colleagues within my department

KS3 I share my work experiences and knowledge with my coworkers

KS4: I try to share my expertise from education or training with other group members in a more effective way

KS5: I show my co-workers how to perform the most difficult part of the work

KS6: I actively answer questions posed by my coworkers

**Intellectual Capital**

**Human Capital**

HC1: employees hold suitable work experience for accomplishing their job successfully in our organization

HC2: employees of our organization have excellent professional skills in their particular jobs and functions

HC3: our organization provides well-designed training programs.

HC4: the employees of our organization often develop new ideas and knowledge

HC5: employees are creative in our organization

**Structural Capital**

SC1: the overall operations procedure of our organization is very efficient.

SC2: our organization responds to changes very quickly

SC3: our organization has an easily accessible information system

SC4: systems and procedures of our organization support innovation

SC5: our organization’s culture and atmosphere are flexible and comfortable

SC6: there is supportive among different departments in our organization

**Relational Capital**

RC1: our organization discovers and solves problems through intimate communication and effective collaboration

RC2: our organization maintains appropriate interactions with its stakeholders

RC3: our organization maintains long-term relationships with customers (importers or merchants)

RC4: our organization has stable and good relationships with the strategic partners
